# Effect of Orbital Decompression on Corneal Topography in Patients with Thyroid Ophthalmopathy

**DOI:** 10.1371/journal.pone.0133612

**Published:** 2015-09-09

**Authors:** Su Ah Kim, Su Kyung Jung, Ji Sun Paik, Suk-Woo Yang

**Affiliations:** Department of Ophthalmology, Seoul St. Mary’s Hospital, College of Medicine, The Catholic University of Korea, Seoul, Korea; Medical College of Soochow University, CHINA

## Abstract

**Objective:**

To evaluate changes in corneal astigmatism in patients undergoing orbital decompression surgery.

**Methods:**

This retrospective, non randomized comparative study involved 42 eyes from 21 patients with thyroid ophthalmopathy who underwent orbital decompression surgery between September 2011 and September 2014. The 42 eyes were divided into three groups: control (9 eyes), two-wall decompression (25 eyes), and three-wall decompression (8 eyes). The control group was defined as the contralateral eyes of nine patients who underwent orbital decompression surgery in only one eye. Corneal topography (Orbscan II), Hertel exophthalmometry, and intraocular pressure were measured at 1 month before and 3 months after surgery. Corneal topographic parameters analyzed were total astigmatism (TA), steepest axis (SA), central corneal thickness (CCT), and anterior chamber depth (ACD).

**Results:**

Exophthalmometry values and intraocular pressure decreased significantly after the decompression surgery. The change (absolute value (|x|) of the difference) in astigmatism at the 3 mm zone was significantly different between the decompression group and the controls (p = 0.025). There was also a significant change in the steepest axis at the 3 mm zone between the decompression group and the controls (p = 0.033). An analysis of relevant changes in astigmatism showed that there was a dominant tendency for incyclotorsion of the steepest axis in eyes that underwent decompression surgery. Using Astig PLOT, the mean surgically induced astigmatism (SIA) was 0.21±0.88 D with an axis of 46±22°, suggesting that decompression surgery did change the corneal shape and induced incyclotorsion of the steepest axis.

**Conclusions:**

There was a significant change in corneal astigmatism after orbital decompression surgery and this change was sufficient to affect the optical function of the cornea. Surgeons and patients should be aware of these changes.

## Introduction

Thyroid-associated ophthalmopathy refers to a pathological autoimmune condition characterized by lymphocytic infiltration and edema of the retrobulbar tissues, resulting in marked thickening and fibrosis of extraocular muscles and orbital fat [1]. In this devastating orbital inflammatory process, T helper 1-type CD4^+^ T cells have been shown to be the dominant inflammatory cells and various cytokines released by these cells stimulate cell proliferation, GAG synthesis, and recruitment of new fat cells [2]. “Watchful waiting” can be one option in minimally symptomatic patients and elective orbital decompression surgery is performed in patients with severe, cosmetically unacceptable proptosis. However, emergency orbital decompression surgery may be needed when blinding symptoms occur, such compressive optic neuropathy due to retro-orbital tissue hypertrophy.

Considerable progress has been made in surgical techniques to achieve both functional and cosmetic success in these patients. A range of surgical techniques, from minimally invasive orbital decompression to two- or three-wall decompression with orbital fat removal, is available [3]. Although many studies have reported the successful management of proptosis or optic nerve compression after orbital decompression surgery, these invasive strategies each have their own risks and unexpected complications. Cerebrospinal fluid (CSF) leakage or permanent new-onset postoperative diplopia are serious complications that may lead to devastating consequences. Thus, a preoperative discussion regarding particular details of any changes that may occur after the surgery is essential. Intensive pre-operative discussions and assessments should be conducted, especially in patients undergoing orbital decompression surgery primarily for cosmetic reasons, and these should include the possibility of diplopia and changes in vision.

Mombaerts et al. showed that Graves’ ophthalmopathy was associated with greater with-the-rule corneal astigmatism and suggested that this was due to fibrosis of the soft tissue in the superolateral orbital region [4]. However, little is known about changes in corneal astigmatism after orbital decompression surgery. Moreover, to our knowledge, no reported study has evaluated changes in corneal astigmatism using corneal topography, which enables the visualization of regional changes in the corneal curvature. Thus, this study was performed to evaluate the refractive and corneal topographic changes following orbital decompression surgery.

## Patients and Methods

Our retrospective study involved 42 eyes from 21 patients who underwent elective orbital decompression surgery for thyroid-associated ophthalmopathy between September 2011 and September 2014 at the oculoplastic division of the Department of Ophthalmology, Seoul St. Mary’s Hospital. Patient records were anonymized and de-identified prior to statistical analysis. This research was conducted according to the principles of the Declaration of Helsinki and was approved by the Seoul St. Mary’s Hospital Institutional Review Board, The Catholic University of Korea (IRB No. KC14RISI0835).

At the initial visit, all subjects underwent comprehensive ophthalmological examinations, including slit-lamp examinations, visual acuity testing, and automated refraction. Corneal topography (Orbscan II; Bausch & Lomb, Rochester, NY), Hertel exophthalmometry, and intraocular pressure were measured at 1 month before and 3 months after surgery. Orbscan II is a scanning slit-beam-based optical reflectance instrument, capable of taking images of anterior and posterior corneal elevation and surface curvature. It combines the scanning slit with Placido-ring videokeratography to obtain the advantages of both and to generate curvature-based (corneal power) maps [5]. At least five scanning procedures were performed for each eye, and the best quality images were chosen for analysis. The topographic map was centered on the corneal apex. Corneal topographic parameters evaluated included total astigmatism (TA) and steepest axis (SA) in the 3- and 5-mm zones, central corneal thickness (CCT), and anterior chamber depth (ACD). A relevant change in astigmatism was defined as a change in cylinder power of at least 0.2 diopter (D) or a relevant rotation of the axis, defined as a change of > 10°, because smaller changes do not usually affect visual acuity [6].

The 42 eyes were divided into three groups: control (9 eyes), two-wall decompression (24 eyes), and three-wall decompression (8 eyes). The control group was defined as the contralateral eyes of nine patients who underwent two- or three-wall orbital decompression surgery in only one eye. All patients underwent orbital decompression surgery due to cosmetically unacceptable proptosis, except one eye that underwent surgery to resolve uncontrolled intraocular pressure. Patients with acute optic neuropathy, previous orbital surgery, and previous corneal disease were excluded. Patients with severe restrictive strabismus who were unable to fixate on the central target, which is required to obtain corneal topography images, were also excluded.

All surgeries were performed under general anesthesia. Local anesthetic (bupivacaine 0.5% with 1:200,000 epinephrine) was injected at the lateral canthus, medial, and lateral peribulbar sites. The nasal mucosa was packed with sterile gauze soaked in a 1:200,000 dilution of epinephrine. All patients underwent two- or three-wall orbital decompression, based on clinical presentation and the degree of proptosis reduction during surgery. Extraconal and intraconal fat excision was also performed. Approximately 3.5 mL of fat were removed with careful hemostasis; the volume was recorded accurately.

## Results

Patient demographics and baseline characteristics are shown in Table 1. The study population comprised 13 females and 8 males. Two-wall decompression was performed in 25 eyes and three-wall decompression in 8 eyes. The mean age of each group was 55.78±8.15 (control), 49.92±13.61 (two-wall decompression), and 39.0±15.97 (three-wall decompression). The average exophthalmometry values and intraocular pressures, measured before decompression surgery, are also shown in Table 1. Both the exophthalmometry values and intraocular pressure decreased significantly after the surgery (both p < 0.001). No statistically significant difference was found in the changes in these values between the two- and three-wall decompression groups (p = 0.8845 and p = 0.3086, respectively).

**Table 1:** Demographics of study patients.

	Control group (*n* = 9)	Two-walled surgery (*n* = 25)	Three-walled surgery (*n* = 8)	*P*-value
**Age**	55.78±8.15	49.92±13.61	39.0±15.97	
**Gender (% female)**	6:3(33.3)	7:18(72)	3:5(62.5)	
**Visual Acuity**				
** Preoperative**	0.92±0.10	0.81±0.19	0.81±0.15	0.1252^†^
** Postoperative**	0.94±0.10	0.94±0.11	0.94±0.10	0.9813^†^
** *P* value**	0.6817*	* **0.0021**	*0.0723	
**Exophthalmometry (mm)**				
** Preoperative**	15.67±2.87	19.56±2.16	21.19±1.81	0.0642^†^
** Postoperative**	15.56±2.60	15.14±2.51	16.13±1.64	0.2901^†^
** Difference**	-0.11±0.93	-4.46±1.70	-5.06±2.21	0.8845^†^
** *P* value**		**<0.0001** *	**0.0003** *	
**IOP (mmHg)**				
** Preoperative**	17.89±2.89	20.16±6.05	19.00±2.45	0.6037^†^
** Postoperative**	17.89±3.66	15.32±3.38	16.25±3.06	0.4938^†^
** Difference**	0±3.571	-4.84±6.22	-2.75±2.60	0.3086^†^
** *P* value**		**0.007** *	**0.0203** *	

Average exophthalmometry values and intraocular pressure, measured before and after surgery, are also shown. Significant P values are indicated in bold.

*Groups were compared with the paired *t*-test.

^†^Groups were compared with the Mann-Whitney U-test (two-tailed).

Pre- and postoperative topographic measurements (astigmatism at the 3-mm and 5-mm zones, steep axis at the 3-mm and 5-mm zones), central corneal thickness (CCT), and anterior chamber depth (ACD) are summarized in Table 2. The differences in these values (values measured after decompression surgery minus values measured preoperatively) and the changes in these values (the absolute value (|x|) of the difference) are also shown in Table 2. The mean overall change in astigmatism at the 3-mm zone was 0.177±0.13 in the control and 0.515±0.426 in the decompression group. Although pre- and post-operative values of astigmatism at the 3-mm zone were not statistically significant within each group (p = 0.7978 and p = 1.00, respectively), the change in astigmatism at 3 mm was significantly different between the decompression and the control groups (p = 0.0250). The mean overall value of the steepest axis at 3 mm before decompression surgery was 94.357±35.82, showing with-the-rule astigmatism. The change in the steepest axis at 3 mm between the decompression and control groups was also significantly different (p = 0.0331). No statistically significant difference was found in the difference in central corneal thickness or anterior chamber depth between the groups.

**Table 2:** Summary of topographic parameters evaluated by the Orbscan (at 3 and 5 mm) in each group.

	All (*n* = 42)	Decompression group (*n* = 33)	Control group (*n* = 9)	*P* value
**Visual Acuity**				
** Preoperative**		0.806±0.166	0.920±0.103	**0.0431** ^**†**^
** Postoperative**		0.934±0.104	0.940±0.097	0.6362^†^
** *P* value**		**<0.001** *	0.681*	
**Astigmatism, 3 mm (D)**				
** Preoperative**	1.33±0.99	1.43±1.07	0.98±0.52	0.2308^†^
** Postoperative**	1.36±0.88	1.46±0.94	0.98±0.50	0.1494^†^
** Difference**		0.03±0.67	0±0.23	0.8958^†^
** Change**		0.52±0.43	0.18±0.13	**0.0250** ^**†**^
** *P* value**	0.7996*	0.7978*	1.000*	
**Steep Axis, 3 mm (°)**				
** Preoperative**	94.36±35.82	92.18±36.73	102.33±32.97	0.4116^†^
** Postoperative**	94.83±38.75	92.36±40.16	103.88±33.58	0.6722^†^
** Difference**		0.18±15.03	1.56±8.41	0.9598^†^
** Change**		12±8.5	5.56±6.23	**0.0331** ^**†**^
** *P* value**	0.3228*	0.3323*	0.848*	
**Astigmatism, 5 mm (D)**				
** Preoperative**	1.41±0.82	1.42±0.79	1.36±0.975	0.8268^†^
** Postoperative**	1.31±0.73	1.36±0.73	1.14±0.716	0.4351^†^
** Difference**		-0.1±0.89	-0.21±0.39	0.6344^†^
** Change**		0.63±0.63	0.3±0.32	0.1423^†^
** *P* value**	0.4508*	0.6853*	0.141*	
**Steep Axis, 5mm (°)**				
** Preoperative**	92.17±049.67	97.42±46.00	72.88±60.37	0.1925^†^
** Postoperative**	91.33±51.92	93.30±52.94	84.11±50.30	0.2577^†^
** Difference**		-4.1±24.2	11.22±25.74	0.7547^†^
** Change**		17.5±17	14.33±23.94	0.4660^†^
** *P* value**	0.1835*	0.3355*	0.227*	
**Central corneal thickness (μm)**				
** Preoperative**	566.52±36.08	563.42±35.58	577.89±37.67	0.2919^†^
** Postoperative**	565.3±43.11	561.15±45.47	580.33±30.50	0.2414^†^
** Difference**		-2.27±22.33	2.44±16.96	0.5603^†^
** Change**				
** *P* value**	0.7015*	0.562*	0.676*	
**Anterior chamber depth (mm)**				
** Pre-operative**	2.58±0.37	2.63±0.36	2.39±0.354	0.0862^†^
** Postoperative**	2.62±0.36	2.65±0.36	2.48±0.365	0.2052^†^
** Difference**		0.03±0.13	0.09±0.22	0.3542^†^
** Change**				
** *P* value**	0.107*	0.273*	0.261*	

The definition of ‘difference’ is subtracting the preoperative value from the postoperative value. The definition of ‘change’ is the absolute value (|x|) of the difference. Significant *P* values are indicated in bold.

*Groups were compared with the paired t-test.

^†^Groups were compared with the Mann-Whitney U-test (two-tailed).

An analysis of relevant changes in astigmatism (change of > 0.2 D) and axis (changes of > 10°) in the two-wall and three-wall decompression groups is shown in Table 3. In Table 3, the values of maximum increase and decrease in astigmatism are also shown. An analysis of relevant astigmatism changes showed that astigmatism changed in 19 of 25 eyes (76%) in the two-wall decompression group and 7 of 8 eyes (88%) in the three-wall decompression group. Similarly, the astigmatism axis changed in 17 of 25 eyes (68%) in the two-wall decompression group and 5 of 8 eyes (63%) in the three-wall decompression group.

**Table 3:** Sub-group analysis of relevant changes in astigmatism power and axis in the two-wall and three-wall decompression groups.

	Two-walled surgery (*n* = 25)	Three-walled surgery (*n* = 8)	*P* value
**Astigmatism Power Change, 3 mm (D)**			
** Number of relevant change cases**	19	7	
** Relevant Power Change**	0.58±0.29	0.76±0.67	0.954*
** Maximum Decrease**	1.2	0.5	
** Maximum Increase**	0.8	2.2	
Astigmatism Axis Change, 3 mm (Degree)			
Number of relevant change cases	17	5	
Relevant Axis Change	14.94±5.61	19.4±13.4	0.606*
Maximum Decrease	30	16	
Maximum Increase	26	43	

*Groups were compared with the Mann-Whitney U-test (two-tailed).

**Fig 1:**
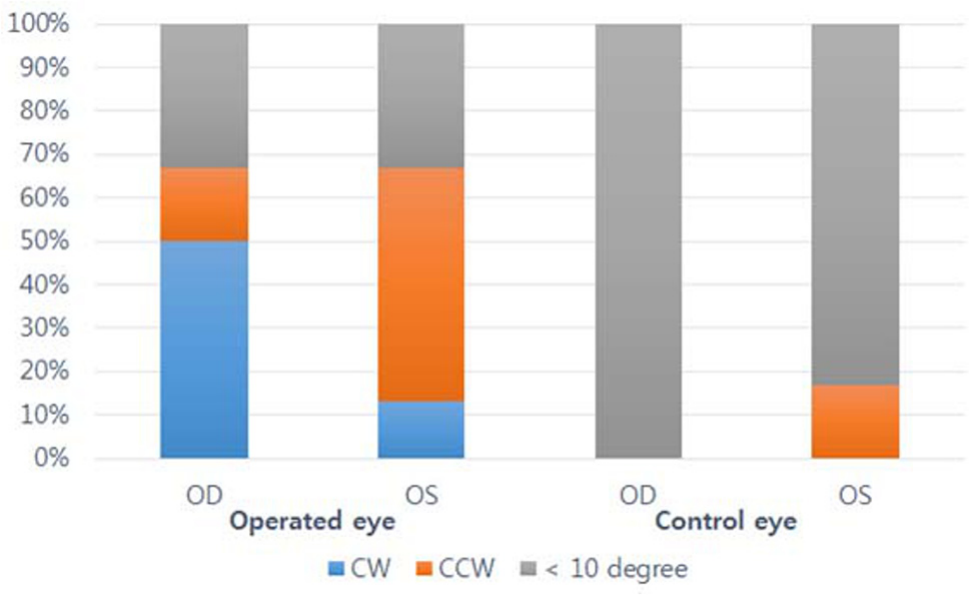
Effect of decompression surgery. This figure demonstrates the direction of the rotation of the steep axis after decompression surgery (CW = clockwise, CCW = counterclockwise, OD = right eye, OS = left eye). (n = 33 in operated eye, n = 9 in control).

Table 4 shows the distribution of all cases depending on the degree and direction of rotation. As shown, among the right eyes that showed relevant axis changes (rotation of axis > 10°), the direction of the change of axis was clockwise in 9 of 11 eyes (82%; Fig 1). In the left eyes, 8 of 10 eyes (80%) rotated counterclockwise. This results indicates that the dominant tendency of the rotation of the steepest axis is incyclotorsion.

**Table 4:** Change and the direction of rotation of the steep axis.

	Operated Right eye (*n* = 18)	Control Right eye (*n* = 3)	Operated Left eye (*n* = 15)	Control Left eye (*n* = 6)
**< 10**	6	3	5	5
**Clockwise**				
**10–20**	8		2	
**> 20**	1			
**Counterclockwise**				
**10–20**	3		6	1
**> 20**			2	

Distribution of all cases depending on the amount of change in the degree of the steep axis and the direction of the rotation of the steep axis. Analyses were performed using topographic parameters measured at the 3-mm zone (Orbscan II).

**Fig 2:**
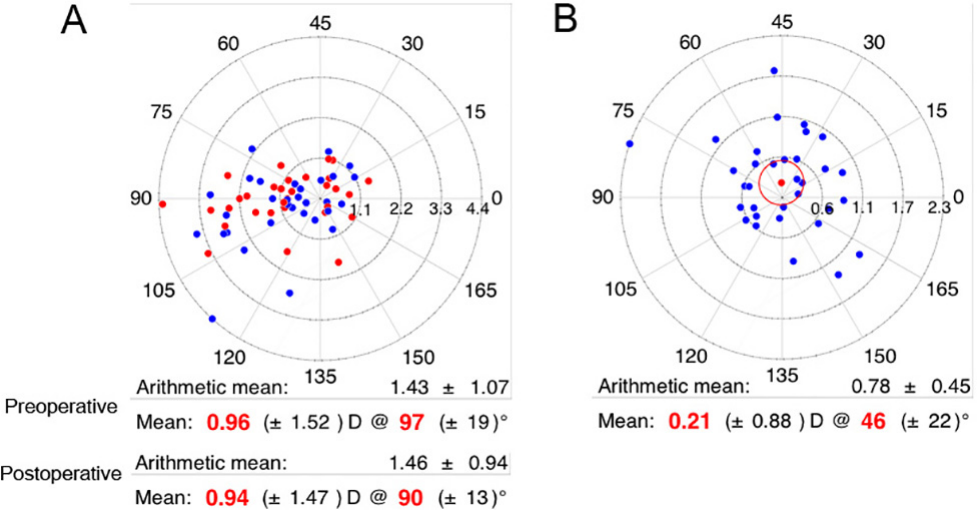
Scatter plots using Astig PLOT. A. Scatter plot of polar astigmatic vectors for preoperative (blue dots) and postoperative (red dots) astigmatism (n = 33). B. Scatter plot of calculated surgically induced astigmatism (SIA) using the mean corneal astigmatism in the 3-mm zone obtained with the Orbscan II (n = 33).

Fig 2A shows a scatter plot of polar astigmatic vectors for pre-operative (blue) and post-operative (red) astigmatism (n = 33). Fig 2B shows the scatter plot of calculated surgically induced astigmatism (SIA) using the mean corneal astigmatism in the 3-mm zone obtained with the Orbscan II (n = 33). The mean pre-operative astigmatism was 0.96±1.52 D, with a mean axis of 97±19° and the mean post-operative astigmatism was 0.94±1.47 D with a mean axis of 90±13°. The mean SIA was 0.21±0.88D with an axis of 46±22°, suggesting that the decompression surgery changed the corneal astigmatism and that a clockwise rotation of the astigmatism axis occurred.

## Discussion

Our data clearly show that decompression surgery in patients with thyroid-associated orbitopathy (TAO) significantly affects corneal curvature, resulting in a shift of the axis towards the incyclotorsion direction. A significant change in corneal curvature after the decompression surgery was only found at the 3-mm zone, not at the 5-mm zone. Furthermore, we showed that decompression surgery affected intraocular pressure and exophthalmometry values.

The cornea is a transparent tissue that exhibits great elastic and viscoelastic properties. Thus, external forces or stress may affect the corneal shape and astigmatism. Lieberman et al. showed that the eyelids distort the corneal shape, just by the lids touching the corneal surface [7]. Moreover, many studies have demonstrated the corneal topographic changes occur not only after surgeries that directly affect the anterior part of the eye, such as a keratectomy but also after surgeries that might be thought to affect only the posterior segment, such as vitreoretinal surgery, including scleral buckling or extraocular muscle surgery [8,9,10]. It is also known that orbital decompression surgery reduces venous congestion, resulting in reduction in eyelid edema and conjunctival chemosis [11]. Our study supports these previous observations in that our data showed significant changes in corneal curvature after orbital decompression surgery.

The primary aim of decompression surgery is to place the eyeball into the ‘proper’ position within the orbit and successful surgery may, at least partially, resolve entropion and lagophthalmos. Thus, it can be hypothesized that orbital decompression surgery may affect corneal shape and eyeball torsion. However, working under this hypothesis, previous studies concluded that corneal astigmatism was not affected by orbital decompression surgery [12,13]. Thus, our results differ significantly from previous observations; there may be several reasons for this. First, the study by Norris et al. used automated refraction and auto-keratometry to measure the corneal shape, whereas we used computerized corneal topography (Orbscan II), which not only has greater accuracy and reproducibility but also enables the visualization of regional changes in the corneal curvature. This advantage of computerized corneal topography enabled the detection of subtle changes in the corneal curvature after the decompression surgery. No reported study has demonstrated changes in corneal shape after orbital decompression surgery using computerized corneal topography. Second, the definition of the “control” group differs in each study. In Norris et al., the controls were patients who underwent intraconal fat removal with or without a one-walled orbital decompression, whereas in our study controls were the contralateral eyes of patients who underwent two- or three-wall orbital decompression surgery in only one eye. This latter definition was considered more appropriate because it can remove changes that may occur during the natural course of the disease itself. Finally, the extent of decompression surgery may differ between the two studies. For example, the amount of fat removed during decompression and the percentage of patients who underwent more extensive surgery, such as a three-wall decompression, differ between the studies.

In our study, Astig PLOT was used to calculate surgically induced astigmatism (SIA). As shown in the Results, the average SIA was 0.21 D with an axis of 46°, indicating that decompression surgery had some effect on corneal astigmatism. Although this amount of SIA may seem small, some previous reports have shown that refractive changes as small as 0.2 D can affect the vision of patients, suggesting that the figures obtained are meaningful. Moreover, the axis of SIA was also affected by decompression surgery and we showed that the preoperative with-the-rule astigmatism was reduced after the surgery. As a previous study^13^ showed that Graves’ ophthalmopathy was associated with greater with-the-rule corneal astigmatism, and was affected by fibrosis of soft tissue in the superolateral orbital region, our data support the hypotheses that (1) lateral wall decompression combined with orbital fat removal may have relieved the superior-lateral compressive force and (2) surgical approaches used in orbital decompression surgery, such as lateral upper eyelid crease incision or lateral canthotomy with cantholysis, may also relieve the compressive force induced by TAO.

Paridaens et al. showed that the proptosis reduction after three-wall decompression surgery and two-wall surgery is significantly different in Graves’ ophthalmolpathy [14]. However, in our study, no significant difference was observed not only in the changes of exophthalmometry values but also in the changes of intraocular pressure between the three-wall and two-wall decompression groups. Considering these differences, further study is needed because more extensive surgery, such as three-wall decompression surgery, has been related to a higher risk of severe complications.

Our study had several limitations. First, the effects of inward or downward deviation of the eyeball on corneal shape were not considered. Inward or downward deviation can be regarded as incyclotorsion or excyclotorsion, respectively. Further study to evaluate the correlation between eyeball deviation and cyclotorsion is warranted. Second, although we consider that defining the controls as the contralateral eyes of patients who underwent two-wall decompression surgery is more precise and appropriate, asymmetric changes between the two eyes that may occur during progression or resolution of the thyroid ophthalmopathy were not considered. For example, subtle asymmetric changes in lid retraction may affect the corneal shape. Finally, the high difference in the number of eyes between three groups may have reduce the power of the statistical tests.

In conclusion, we have demonstrated that a significant change in corneal curvature occurs after orbital decompression surgery in patients with TAO. The amount of this change in corneal curvature is sufficient to affect the optical function of the cornea in these patients. A detailed preoperative consultation is essential for all patients undergoing orbital decompression surgery.

## Supporting Information

S1 Data Astigmatism Analysis. Contains original data sets which were used for analysis.
(XLSX)

## References

[1] Yang D, Hiromatsu Y, Hoshino T, Inoue Y, Itoh K, Nonaka K (1999) Dominant infiltration of T(H)1-type CD4+ T cells at the retrobulbar space of patients with thyroid-associated ophthalmopathy. Thyroid 9:305–310. 10211609 10.1089/thy.1999.9.305

[2] Heufelder AE (2000) Pathogenesis of ophthalmopathy in autoimmune thyroid disease. Rev Endocr Metab Disord 1:87–95. 11704996 10.1023/a:1010020621687

[3] Ben Simon GJ, Schwarcz RM, Mansury AM, Wang L, McCann JD, Goldberg RA (2005) Minimally invasive orbital decompression: local anesthesia and hand-carved bone. Arch Ophthalmol 123:1671–1675. 16344438 10.1001/archopht.123.12.1671

[4] Mombaerts I, Vandelanotte S, Koornneef L (2006) Corneal astigmatism in Graves' ophthalmopathy. Eye 20:440–446. 15846381 10.1038/sj.eye.6701898

[5] Orbscan II (2002) Orbscan IIz, operator’s manual (version 3.12) Bausch and Lomb, Salt Lake City, pp 8–15.

[6] Zinkernagel MS, Ebneter A, Ammann-Rauch D (2007) Effect of upper eyelid surgery on corneal topography. Arch Ophthalmol 125:1610–1612. 18071108 10.1001/archopht.125.12.1610

[7] Lieberman DM, Grierson JW (2000) The Lids Influence on Corneal Shape. Cornea 19: 336–342. 10832695 10.1097/00003226-200005000-00016

[8] Kwitko S, Sawusch MR, McDonnell PJ (1991) Effect of extraocular muscle surgery on corneal topography. Arch Ophthalmol 109:873–878. 2043078 10.1001/archopht.1991.01080060137042

[9] Nardi M, Rizzo S, Pellegrini G, Lepri A (1997) Effects of strabismus surgery on corneal topography. J Pediatr Ophthalmol Strabismus 34:244–246. 9253740 10.3928/0191-3913-19970701-13

[10] Hainsworth DP, Bierly JR, Schmeisser ET, Baker RS (1999) Corneal topographic changes after extraocular muscle surgery. J AAPOS 3:80–86. 10221799 10.1016/s1091-8531(99)70074-1

[11] Wakelkamp IM, Baldeschi L, Saeed P (2005) Surgical or medical decompression as a first-line treatment of optic neuropathy in Graves' ophthalmopathy? A randomized controlled trial. Clinical Endocrinology 63:323–328. 16117821 10.1111/j.1365-2265.2005.02345.x

[12] Norris JH, Ross JJ, Kazim M (2012) The effect of orbital decompression surgery on refraction and intraocular pressure in patients with thyroid orbitopathy. Eye 26:535–543. doi: 10.1038/eye.2011.362 22261739 10.1038/eye.2011.362 PMC3325579

[13] Mombaerts I, Vandelanotte S, Koornneef L (2006) Corneal astigmatism in Graves; ophthalmopathy. Eye 20:440–446. 15846381 10.1038/sj.eye.6701898

[14] Paridaens D, Lie A, Grootendorst RJ, van den Bosch WA (2006) Efficacy and side effects of ‘swinging eyelid’orbital decompression in Graves’orbitopathy: a proposal for standardized evaluation of diplopia. Eye 20:154–162. 15746952 10.1038/sj.eye.6701827

